# ‘Thank you for loving me’: A qualitative study on perceptions of gratitude and their effects in palliative care patients and relatives

**DOI:** 10.1177/02692163231207495

**Published:** 2023-11-09

**Authors:** Emmanuelle Poncin, Emilie Bovet, Emmanuel Tamches, Boris Cantin, Josiane Pralong, Betty Althaus, Gian Domenico Borasio, Mathieu Bernard

**Affiliations:** 1 Palliative and Supportive Care Service, Lausanne University Hospital, University of Lausanne, Lausanne, Vaud, Switzerland; 2 La Source School of Nursing, HES-SO, Lausanne, Switzerland; 3 Haute École de Santé Vaud (HESAV), Haute école spécialisée de Suisse occidentale (HES-SO), Lausanne, Switzerland; 4 Palliative Care Center, Fribourg Hospital, Fribourg, Switzerland; 5 Rive Neuve Foundation, Blonay, Switzerland

**Keywords:** Gratitude, palliative care, qualitative research, psychology, positive, discourse analysis

## Abstract

**Background:**

Empirical studies suggest that gratitude positively influence the quality of life of palliative patients and relatives. However, the literature is marked by a lack of conceptual clarity about what gratitude is and whether it can bring about individual and social benefits.

**Aim:**

This paper explores how palliative care patients and relatives understand gratitude, how discursive representations of gratitude may affect their positions, perceptions and relations, and how to conceptualise gratitude in the palliative context.

**Design:**

We examine 33 gratitude letters written by patients and relatives and 25 semi-structured interviews conducted as part of a pilot gratitude intervention study. We use a qualitative approach, thematic analysis, within a conceptual framework of discourse analysis.

**Settings/participants:**

Data were collected from 23 patients and 13 relatives recruited through three hospital palliative care services in French-speaking Switzerland.

**Results:**

Participants articulate gratitude in five ways: (1) appreciating others; (2) love; (3) need to reciprocate; (4) appreciating the little things; (5) solace amid serious illness. While some of these representations are sources of positive emotions and outlook, wellbeing and hope, others may confirm self-perceptions of powerlessness and burden. These results support a tridimensional conceptualisation of gratitude in palliative care as source of individual benefits, valuing closest relationships and moral obligation.

**Conclusion:**

Our study suggests that gratitude is a key to a good (end of) life, whilst highlighting potential negative effects. It could help healthcare professionals to better understand what gratitude means to patients and relatives, which may facilitate awareness and fostering of gratitude in palliative care.

***i) What is already known about the topic?***

- Gratitude is associated with greater wellbeing and reduced psychological distress in palliative care patients.
- Gratitude interventions may help palliative care patients and their relatives experience emotional wellbeing, personal growth and improved relationships.

***ii) What this paper adds***

- Palliative care patients represent gratitude as love and loved ones, which may give rise to positive emotions and outlook, wellbeing and may strengthen their will to live.
- Some patients articulate gratitude as a need to reciprocate in relationships of care dependence.
- For relatives, gratitude represents solace in the midst of serious illness.

***iii) Implications for practice, theory or policy***

- Understanding the perceptions of gratitude and their effects could help healthcare professionals to better integrate gratitude awareness and fostering into psychosocial therapeutic approaches in palliative care.
- Following the *find-remind-and-bind* theory, our study suggests that the main function of gratitude is to sustain one’s closest relationships.
- Gratitude may have negative emotional effects in relationships where the power balance tips towards the benefactor or family carer.

## Introduction

The advent of positive psychology in the 21st century has given rise to mounting interest in the concept of gratitude. Research has shown that gratitude is associated with greater wellbeing and reduced psychological distress in the general population^1,2^ and in clinical populations, notably breast cancer patients^3,4^ and palliative care patients.^5,6^ In this context, interventions were designed to foster gratitude, usually by journaling about what one feels grateful for or writing gratitude letters.^7^ Studies found that gratitude interventions help improve aspects such as daily psychological functioning, perceived support, use of adaptive coping and spiritual wellbeing, and lessen fear of death, anxiety and depression in cancer patients.^8–10^

In palliative care, our research team led a pilot gratitude intervention for patients and their family carers, based on writing a gratitude letter to each other and sharing it.^11^ Quantitative results showed a reduction in psychological distress for carers, and qualitative analyses highlighted that the intervention could help patients and carers to experience emotional wellbeing, personal growth and improved relationships.

While empirical studies suggest that gratitude may positively influence the quality of life of palliative patients and their relatives,^5,6^ the wider literature is marked by controversies surrounding the concept of gratitude – what it is, how it manifests, and whether and how it can bring about individual and social benefits.^12^ In psychology, for instance, researchers have alternately depicted gratitude as the act of showing thanks, the emotional response to a perceived benefit, or a trait or predisposition towards ‘noticing and appreciating the positive in the world’.^1,2,13^ Gratitude has been variously characterised as a moral virtue, a moral duty, an expression of humility, a character strength, a catalyst for prosocial actions and behaviours and a source of individual benefits.^13–15^

To add insights on the nature and action mechanisms of gratitude, this paper examines the discourse of palliative patients and their family carers on gratitude. Our aims are to: (a) explore how patients and relatives represent gratitude; (b) understand how these representations may affect patients and relatives’ positions, perceptions and relations; and (c) consider how to best conceptualise gratitude in the palliative care context, based on the above findings. At the clinical level, shedding some light on the perceptions, experiences and effects of gratitude could help to better apprehend and target gratitude interventions (as self-help or as part of supported therapy), which could in turn participate in improving their efficacy and relevance.

## Methods

### Study design

This paper examines material produced as part of a pilot study that sought to assess the feasibility, acceptability and effects of a gratitude letter-writing intervention in palliative care.^11^ We analysed gratitude letters and semi-structured interviews with palliative patients and relatives who took part in the intervention. We adopted a discourse analysis framework to make sense of participants’ representations of gratitude and their effects, and conducted inductive thematic analysis of our material.

### Methodological orientation

This paper is rooted in the social constructivist tradition, which seeks to understand how people perceive and experience the social world, and regards such experiences and perceptions as subjective constructs negotiated at the level of the individual and the group (e.g. influenced by social, economic, cultural and political dynamics).^16^ As such, we propose to give insights into the unique, contextualised experiences and perceptions of our participants, which we believe can help to understand the experiences and perceptions of others in similar situations.

Our main object of analysis is discourse, understood as a fluid and context-specific set of meanings, representations, positions and relations. In this view, the key characteristic of discourse is its performativity: discourse does not simply say something about the world; it affects it through the production of meanings, identities and relations.^17–20^ The ways in which discourse affects the social world – functioning as a form of social action instead of merely describing – are referred to as performative effects.

### Settings

Recruitment for the study took place between November 2018 and March 2020 in three public inpatient hospital palliative care services in French-speaking Switzerland. The study was approved by the Ethics Committee of the Canton of Vaud (n°2018-01309).

### Population

Patients’ eligibility criteria were: (i) age >18, (ii) progressive illness with reduced life expectancy, (iii) enrolled in palliative care, (iv) clinical state enabling the person to take part in research, (v) no total social isolation, (vi) no significant cognitive or psychiatric disorders and (vii) no severe communication problems. Carers’ eligibility criteria were: (i) age >18, (ii) no significant psychiatric or cognitive disorders and (iii) no severe communication problems.

### Sampling/recruitment

We used a convenience sampling strategy. Care teams in participating institutions identified eligible patients and asked for their permission to be contacted by a researcher. Prospective participants were informed about the study during a face-to-face meeting with a researcher (BA or EP) and provided with a study information document. They were also asked to identify a family carer with whom they wished to perform the intervention (i.e. write and share a gratitude letter). The later were informed about the study orally (in person or by phone) and in writing. Prospective participants were informed they would be offered professional psychological support if they were to experience difficulties or distress during the study. All participants provided oral and written informed consent.

### Data collection

We conducted 25 semi-structured interviews 5–20 days after the gratitude letter-writing intervention, asking about the meaning, personal experiences and sources of gratitude, as detailed in Table 1 (see Bernard et al.^11^ for the full interview guide).

**Table 1.** Interview guide – Questions on representations and experiences of gratitude.

What does the term ‘gratitude’ mean to you?
What are your personal experiences with gratitude?
Could you give me some examples?
Do you usually express your gratitude?
If so, how do you express it?
What or who do you feel grateful towards?

All interviews were conducted in French. They took place in palliative care services for inpatients, and in participants’ homes for discharged patients and relatives. Four of the interviews were conducted with both the patient and relative; 21 took place with one participant and a researcher present. All interviews were audio-recorded and transcribed verbatim. No repeat interviews were carried out. The researcher who informed potential participants about the study conducted the corresponding interviews. They had no contact with participants prior to this research. Both interviewers had experience and training in qualitative methodologies (EP, PhD in Government) and psychology (BA, MSc Psychology). They presented themselves as researchers, underlining their independence from participants’ care teams.

We also examine 33 gratitude letters, which palliative patients and family carers wrote to each other as part of the pilot gratitude intervention, following instructions to:

take a moment to think back about the past few years and remember when [your relative] did something for you, for which you feel extremely grateful. Think about the impact that he/she had on your life. Now, take a moment to write a letter to this person.

Next, and only if you wish to do so, you will have the possibility to read her/him the letter, to ask her/him to read it in front of you, or to let her/him read it alone.

The instructions further clarified that ‘*Everything that you write will remain strictly confidential, unless you give your permission to a researcher to retain a copy of your letter for the purpose of this study*’. Participants could decide whether to share their letter with the addressee at any point after writing and in the way of their choosing.^11^

As this research was conducted as part of a larger study with a predetermined sample size of 30 participants, based on recommendations for pilot studies^11^, data saturation was not discussed.

### Data analysis

We used discourse analysis as a broad conceptual framework to direct the analytical lens towards questions of how discourse shapes (and is shaped by) specific perceptions, identities and relations. To identify patterns and trends characterising participants’ discourse, we used inductive thematic analysis,^21^ an iterative process through which materials are read and coded, codes are compared to generate themes and themes are further refined by re-reading materials and further coding. Two researchers (EB, EP) familiarised themselves with the entire body of data. They independently coded six randomly selected letters and developed their own codebooks. At this stage, coding was open and detailed, as the aim was to capture the richness of the letters. After sharing their results with each other and agreeing on a flexible coding structure, EP continued coding letters and developing themes. EB performed a final crosscheck. MAXQDA was used for this analysis.

We further refined the research questions guiding the present enquiry before turning to the interview transcripts, which were organised in an Excel file to compare participant’s definitions, sources, experiences, tendency to express (or not) and effects of gratitude. These data were then coded and codes pertaining to interview data and letters were compared to develop final themes (see coding tree in supplementary material 1). The analysis was performed on the original French language materials. The main author (EP), a native French speaker fluent in English, translated quotes when writing the article. The translations were then discussed with a native English speaker. We have included the original French quotes as supplementary material 2, as translation always carries the risk of misconstructing participants’ original meanings^22^ – although viewed through the lens of constructivism, meanings are never fixed and necessarily subjected to processes of inner and outer translation of an intangible ‘being’ into expressions of conscious representations.^23^

## Results

### Participants

This paper examines materials produced by 23 patients and 13 relatives – for the pilot study recruitment flowchart, see Bernard et al.^11^ Fifteen patients participated in the interviews and shared their gratitude letter with the researchers, 6 shared their letter only and 2 did the interview only; 11 relatives participated in the interviews and shared their letter, 1 shared a letter only and 1 participated in the interview only. All participants who wrote a letter agreed to share a copy with the research team during their post-intervention interview. Participants addressed their letters to their spouse or partner (15 participants), sibling (3 participants), friend (3 participants), child (3 participants), parent, (1 participant), and son in law (1 participant). Despite instructions to select a family carer, some addressed their letters to a professional carer (3 participants) or patient (2 participants). As presented in Table 2, the mean patient age was 66, 70% were female, 43% were married or in a registered partnership, 65% had a cancer diagnosis and 56% were capable of limited or no self-care. The mean age for relatives was 61, 85% were female and 69% were married or in a registered partnership, as shown in Table 3.

**Table 2.** Patients’ characteristics (*n* = 23).

Variables	Value	(%)
Age		
Mean	66	
Standard deviation	12	
Sex		
Female	16	70
Male	7	30
Mother tongue		
French	17	74
Spanish	4	17
Italian	2	9
Marital status		
Single	4	17
Married or in a registered partnership	10	43
Divorced or separated	6	26
Widow	3	13
Primary diagnosis		
Cancer	15	65
Other	7	30
Missing data	1	4
ECOG performance status		
1. Restricted in physically strenuous activity but ambulatory and able to carry out work of a light or sedentary nature	3	13
2. Ambulatory and capable of all selfcare but unable to carry out any work activities; up and about more than 50% waking hours	6	26
3. Capable of only limited selfcare; confined to bed or chair more than 50% waking hours	10	43
4. Completely disabled; cannot carry out any selfcare; totally confined to bed or chair	3	13
Missing data	1	4

ECOG: eastern cooperative oncology group.

**Table 3.** Relatives’ characteristics (*n* = 13).

Variables	Value	(%)
Age		
Mean	61	
Standard deviation	15	
Sex		
Female	11	85
Male	2	15
Mother tongue		
French	13	100
Marital status		
Single	3	23
Married or in a registered partnership	9	69
Divorced or separated	1	8

### Gratitude: Representations and performativity

**Figure 1.**
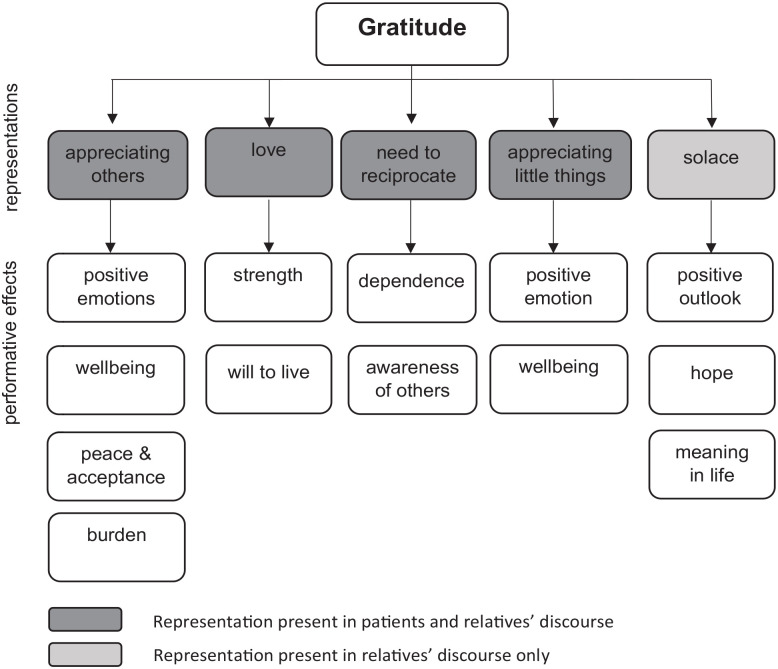
Participants’ representations of gratitude and their performative effects.

We identified five main ways in which participants articulate gratitude, woven through and intertwined in their narratives. We describe each in turn, seeking to highlight their performative effects, as shown in Figure 1.

In the paragraphs below, participants are identified by a code. Members of a same dyad are identified by the same code – followed by no specific mention for patients, or by the mention ‘-relative’; and by no specific mention for interview data, or by the mention ‘-letter’.

#### Appreciating other people

In interviews, participants explained that they are thankful for their partners, family, friends or neighbours for being ‘here for me at all times’ (Z081) and ‘being so great’ (X015). Some relatives defined gratitude along interpersonal lines, as a ‘recognition of what the other can bring’ (X015-relative). Some patients went further, personalising gratitude as the person by their side: ‘the ultimate gratitude, for me, it was [my wife]’ (Z159).

In letters, participants also thanked their loved ones for their presence and for ‘supporting us [. . .] everyday’ (X015-relative), their quality (‘I don’t know how to thank you for all the good that you have done me, and your kindness and your goodness’, Y039-letter), or for concrete endeavours such as ‘all the tasks around the house’ (Y042-letter). Some patients framed this appreciation of others as a source of happiness (‘she [my wife] is the ray of sun in the darkness of the illness, without her presence my life is dull’, Y037-letter), ‘acceptance’ of the illness (Y008), and positive emotions even at the most difficult times (‘after the operation, it was my parents that were there all of a sudden at my bedside to hold my hand [. . .] so it was, yeah, it was a good feeling for me, even with all the pain’, Z067). A patient also explained that: ‘If I die tomorrow because of this illness, somehow I will be at peace with myself because I thanked my mother [. . .] verbalising was really salutary’ (X015).

Appreciating others was at times tainted by a feeling of burden, as some patients said they ‘can’t give much back in return’ (Y037) or described themselves as a cause of worry:

I know how tired you are, I know how much time you spend taking care of me. I know I am a source of worry for you and, even when you are exhausted, whether physically or morally, you are here. For me and only me. (Z057-letter)

By contrast to patients, some family carers explained that gratitude is linked to ‘all the support we can bring each other, all those beautiful things that happen between humans’ (Z172-relative).

#### Love

In participants’ discourse, love was often identified as the main object of gratitude (‘Thank you for loving me’, Y029-letter) and defined as ‘an extraordinary feeling. Very close to love’ (Z144-relative). Some patients defined gratitude as love itself, as ‘an act of love’ (X025) or ‘above all to love the person’ (Y037). Several participants also expressed greater depth and awareness of love in the context of serious illness, which ‘brought us even closer to each other [. . .] and showed us that we really love each other’ (Z081-letter). A relative wrote to her husband:

The illness has befallen you, us, without any warning, turning everything upside down, rendering everything futile, superfluous, without any interest, everything but one essential thing: Love. The love that these terrible circumstances have, to my eyes, strengthened throughout these terrible moral and physical challenges that you overcame and that you are still facing today, so bravely. (X015-relative-letter)

Patients further described this ‘gratitude as love’ as a ‘treasure’ (Z057-letter), which protects them (‘What would I have done in the midst of all these dangers without your love and your wisdom?’, X009-letter) and gives them the strength to keep going and a will to live (‘I am hanging on and I want to continue for the longest time possible to be with you and to be loved by you’, Y043-letter).

#### Need to reciprocate

whereby gratitude is articulated as something that *must* be expressed – by ‘saying “thank you”’ (Z272; Y042) or by ‘thanking through gestures as much as through words’ (X015). Here, patients’ deepened awareness of gratitude appears to be tied to changing relationships in the face of reduced autonomy and care dependence:

Interviewer: Was it [gratitude] something you were already aware of [before the intervention]?

Relative: No, not really

Patient: I was [. . .]

Relative: Of course my husband depends on me, so . . .

Patient: You could have placed me . . . anywhere. (X009)

The link between gratitude and a sense of dependence was directly expressed by a participant, who explained: ‘I express my gratitude [. . .] I do it now because I’m handicapped, if you will. So everything that happens to me is gratitude’ (Z159). In such context, gratitude was presented as taking on a deeper meaning, as illustrated by a patient who wrote:

Since then [the illness], I really learned the signification of the words ‘thank you’ and of gratitude. It’s not always easy as I was used to manage alone, to decide alone in my professional life and suddenly, overnight, I become dependent on you, on the kids, the doctors, nurses, medicine, care . . . (X015-letter)

In parallel, a relative conveyed an expectation that her actions be reciprocated by her husband’s expressions of gratitude:

Interviewer: And do you usually express your feeling of gratefulness or gratitude [. . .]?

Relative: (laughs) No need (laughs) . . . No, well, it’s part of . . .

Patient: Who are you taking about? You tell me ‘I did this, I don’t have to do it . . .’

Relative: No, it’s true. Sometimes I point certain things out to him [my husband] [. . .] I tell him: ‘You have to realise that I am not obliged to do that’ but I do it because I love my husband and I want him to feel as good as possible. (X009)

Gratitude as need to reciprocate may thus be framed as an opportunity for deepened awareness of others or as a necessity in relationships of care dependence, which may reaffirm the self-perception of being a powerless patient. A relative acknowledged this ambivalent side of gratitude, defining the concept as ‘thanking also as best we can, let’s say, not burdening the other too much [. . .] I think that it’s also a form of gratitude, to try to make the other feel good as well’ (Z057-relative).

#### Appreciating the little things

Some people highlighted that appreciating the little things in life is the essence of gratitude – whether it is linked with other people (‘the long chats on the phone’, X025-letter) or nature (‘when I [. . .] see this landscape, well . . . I am filled with gratitude’, Z057-relative). Patients who embraced this conception of gratitude explained that it brought them ‘some wellbeing here and there’ (Z057), or made them feel ‘lucky’ (Y037; X025-letter). Likewise, a relative explained that:

There are . . . ah breaths of (exhales) . . . of happiness that come from I don’t know where. And I think that it’s linked with gratitude. When I enter my garden or I go stroll in the countryside, I feel such a sense of wellbeing that I have the impression that there’s an exchange. (X015-relative)

For some participants, this conception of gratitude was closely linked with living in and appreciating the present moment, as a relative who wrote to her sister: ‘Every day is a pretty miracle that I savour next to you: THANK YOU’ (Z080-relative-letter). A similar view was expressed by a patient, who explained that by ‘setting the bar a little lower’, ‘just being able to get out of bed, get dressed without help, shower, get ready, meet people [. . .] all these, to me, are reasons to be grateful’ (Z172). She further stated that:

to be grateful also helps cheer you up and I think that if we all made a little more effort to think about it, well maybe we would manage to improve our quality of life. [. . .] we must hang on to every little thing that can show us that it’s still worth it and that’s what I told myself also when I learned that, on the face of it, what I have is incurable.

#### Solace in the midst of serious illness

In interview, some relatives defined gratitude as ‘a sort of grace’ (Z159-relative), a way to ‘live together as best we can and . . . and to take things, we try, positively’ (X009-relative). This gratitude was, for some, directed at their loved ones’ courage throughout the illness (‘the way in which you overcome the illness also gives me a reason to thank you’, X015-relative-letter). For others, shared moments of happiness were the objects of gratitude:

Thank you for sharing in the everyday difficulties that the illness is imposing on you, sweet moments of tenderness and Love, pretty smiles and bursts of laughter, a craziness that belongs to us alone. (Z080-relative-letter)

This concept of gratitude was linked with hope for a relative (‘Today I thank you for accepting to fight, to combat this cancer so that we can still have beautiful years before us’, Z057-relative-letter), and with a sense of purpose for another (‘you are accepting what happened to you better than I do, in such a way that helping you isn’t a duty for me, but gives meaning to my life’, Z008-relative-letter). More generally, the conceptualisation of gratitude as solace is closely intertwined with its performative effects, as gratitude is understood as a way to live, apprehending things in a positive light, which helps people adopt a positive outlook on life.

### Conceptualising gratitude

**Figure 2.**
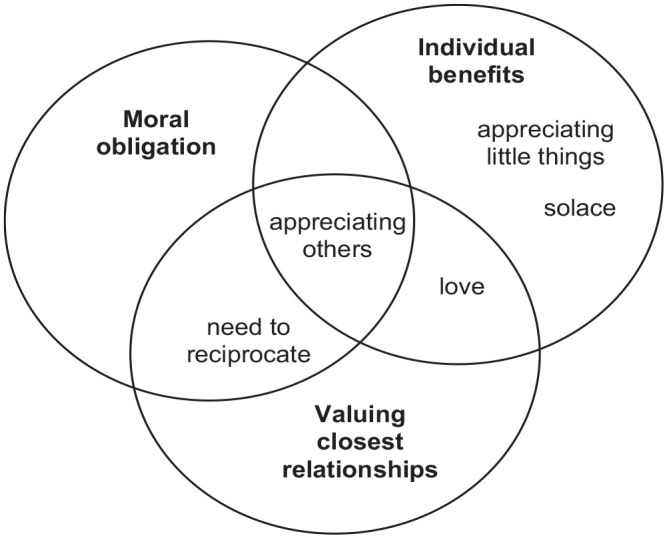
Conceptualising gratitude in the palliative care context.

The above results may be summarised through a tridimensional conceptualisation of gratitude in the palliative care context, illustrated in Figure 2. The first dimension is gratitude as source of individual benefits, including positive emotions, wellbeing, strength, peace and acceptance, will to live, positive outlook, hope and meaning in life – all of which could be glimpsed in participants’ representations of gratitude as appreciation of others, appreciating the little things, love and solace. The second dimension of gratitude is valuing one’s closest relationships, with participants equating gratitude with love, representing it as appreciating close friends and relatives, personalising gratitude as the person by the side and gaining deepener awareness of others. The third dimension is that of moral obligation in relations of care dependence, where gratitude is presented as necessary to reciprocate others’ favours, or where appreciating others may confirm or exacerbate feelings of burden.

## Discussion

### Representations of gratitude in palliative patients and relatives’ discourse

Our study supports the view of gratitude in the palliative care context as primarily a trait or tendency to appreciate the positive in one’s life. We identified five ways in which palliative care patients and their relatives articulate gratitude: (1) appreciating others; (2) love; (3) need to reciprocate; (4) appreciating the little things; and (5) solace – with several representations cohabiting in the discourse of most participants. Some conceptualisations were common to patients and relatives, with similar foci (appreciating the little things) or variations in intensity and focus (love, appreciating others, need to reciprocate). Gratitude as solace was mainly populating relatives’ discourse, articulated along the lines of being thankful for the other’s courageous fight, acceptance and will to live.

Themes 1, 4 and 5 echo aspects of trait gratitude inventoried by Wood and colleagues,^1^ namely ‘appreciation of other people’ and ‘appreciation rising from understanding life is short’ – a qualification particularly relevant to the palliative care context. If some participants linked appreciating the little things with life-limiting illness, others focused on their appreciation, regardless of a sense of finality or acute consciousness of one’s mortality.

Gratitude as love finds parallels in a qualitative study that identifies love as part of a ‘cascade of emotion labels that attempt to characterise the lived experience of gratitude’.^24^ Our patients equated rather than compared the two constructs, as they did when personalising gratitude as the person by their side. This suggests that faced with serious illness, people strongly associate gratitude with love and loved ones, who are primary factors contributing to meaning and quality of life for palliative patients.^25^

Gratitude as need to reciprocate echoes qualitative work on care dependence at the end of life, which entails ‘a radical change’ in the ways in which people view themselves and others.^26^ In this context, patients may experience intertwined feelings of burden and gratitude, which may trigger a desire or need to reciprocate.^27^ This theme can also be apprehended against the backdrop of recent empirical work highlighting that ‘receiving favours is a mixed blessing’, underlining that ‘favours should elicit more indebtedness to the extent that they increase the level of inequity’ in a relationship.^28^ As such, the need to reciprocate reported by some patients may be linked with (and participate to) a sense of widening inequalities in their relationships – This may lead people to experience expressions of gratitude as sources of shame, frustration and as exacerbating a feeling of powerlessness, as illustrated in the literature on informal or charitable care relations.^29,30^

### Gratitude discourse: Performativity and conceptualisation

Our analysis suggests that discursive constructs of gratitude have specific performative effects on individuals’ positions, perceptions and relations. Gratitude as appreciating the little things, love and solace may give rise to positive emotions and outlook, wellbeing and may strengthen patients’ will to live. Gratitude as appreciating others may also trigger positive emotions, peace, acceptance and wellbeing, just as it may intensify self-perceptions of burden. Lastly, gratitude as need to reciprocate may represent an opportunity for deepening one’s awareness of others. It may also be experienced as a necessity in relationships of care dependence, as the only way to return others’ favours, which may reinforce the self-perception of being a powerless patient.

The above results support a tridimensional conceptualisation of gratitude in the palliative care context as source of individual benefits, valuing closest relationships and moral obligation. Studies on gratitude in palliative care professionals have highlighted trends that parallel our findings in terms of individual benefits. Expressions of gratitude from patients and families were shown to improve the mood of professionals, encourage them to go on, act as a source of support in difficult times and reaffirm the meaning of their work.^31–33^ These studies did not uncover negative effects of gratitude as moral obligation. Such effects may be particularly salient in relations where the power balance tips towards the ‘benefactor’ or ‘carer’ – a point put forward in the literature on the ‘dark side’ of gratitude^34^ and illustrated in a body of work on informal care relations and health interventions in developing countries.^30^

In turn, empirical and theoretical studies have proposed that gratitude primarily serves a social function of building new relationships^35^ or strengthening one’s closest relationships.^36^ Illustrating the later proposition, the *find-remind-and-bind* theory proposes that the main function of gratitude is ‘sustaining the most important relationships of our lives’.^36^ Our study provides support for this theory, with participants expressing deepened bonds of love with their partners, children, parents, siblings or close friends – a particularly important point insofar as older patients identify their loved ones as the primary contributors to their will to live.^37^

### Limitations

Our participating patients had to be well enough to take part in this research and usually manifested an initial interest in gratitude. As such, their views might not reflect that of other palliative patients and family carers. Moreover, this paper focuses on discourse produced for the purpose of research, rather than on ‘naturally-occurring’ discourse, which is the focus of traditional discourse analyses. The gaze of the researcher, whether direct or implicit, thus participated in creating the participants’ discourse. However, we do not regard this as overly problematic, as our epistemological stance is that all discourse is mediated and involves interpretation. Lastly, although the present work self-identifies as social constructivist, it is part of a broader mixed methods pilot study that did not explore the complex social, cultural and political dynamics that may have enabled us to understand our participants’ positions and relations in greater depths.

On a reflexive note, we acknowledge that our data was influenced by the ways in which we presented ourselves and interacted with participants, just as our analysis was guided by our subjectivities. As such, it is important to regard our materials and results as contextualised and negotiated productions.

## Conclusions

In the discourse of our participants, gratitude can mostly be conceptualised as a trait, oscillating between gratitude as a source of individual benefits, valuing closest relationships and moral obligation. Our study supports a view of gratitude as a key to a ‘good life’,^38^ whereby one is fully able to love and appreciate life in general and other people in particular. It also highlights that gratitude has ambivalent aspects, insofar as it may be intertwined with feelings of burden and self-perceptions of dependence and powerlessness. Our analysis could help therapists and other healthcare professionals to better understand what gratitude means to palliative care patients and their relatives and what it may imply, in terms of how they view themselves and others.

Future studies on gratitude in palliative care could build upon our categories to assess their relevance to other socio-cultural contexts, paying particular attention to the performativity of gratitude discourse to better apprehend its effects. This could in turn pave the way for a better integration of gratitude awareness and fostering into psychosocial therapeutic approaches for palliative care patients and their families.

## Supplemental Material

sj-pdf-1-pmj-10.1177_02692163231207495 – Supplemental material for ‘Thank you for loving me’: A qualitative study on perceptions of gratitude and their effects in palliative care patients and relatives Click here for additional data file. Supplemental material, sj-pdf-1-pmj-10.1177_02692163231207495 for ‘Thank you for loving me’: A qualitative study on perceptions of gratitude and their effects in palliative care patients and relatives by Emmanuelle Poncin, Emilie Bovet, Emmanuel Tamches, Boris Cantin, Josiane Pralong, Betty Althaus, Gian Domenico Borasio and Mathieu Bernard in Palliative Medicine

sj-pdf-2-pmj-10.1177_02692163231207495 – Supplemental material for ‘Thank you for loving me’: A qualitative study on perceptions of gratitude and their effects in palliative care patients and relatives Click here for additional data file. Supplemental material, sj-pdf-2-pmj-10.1177_02692163231207495 for ‘Thank you for loving me’: A qualitative study on perceptions of gratitude and their effects in palliative care patients and relatives by Emmanuelle Poncin, Emilie Bovet, Emmanuel Tamches, Boris Cantin, Josiane Pralong, Betty Althaus, Gian Domenico Borasio and Mathieu Bernard in Palliative Medicine
