# Supplementary figures and images for: ‘Thank you for loving me’: A qualitative study on perceptions of gratitude and their effects in palliative care patients and relatives
Source: Palliat Med. 2023 Nov 9;38(1):110–20. doi: 10.1177/02692163231207495 (PMC10798025; doi:10.1177/02692163231207495)

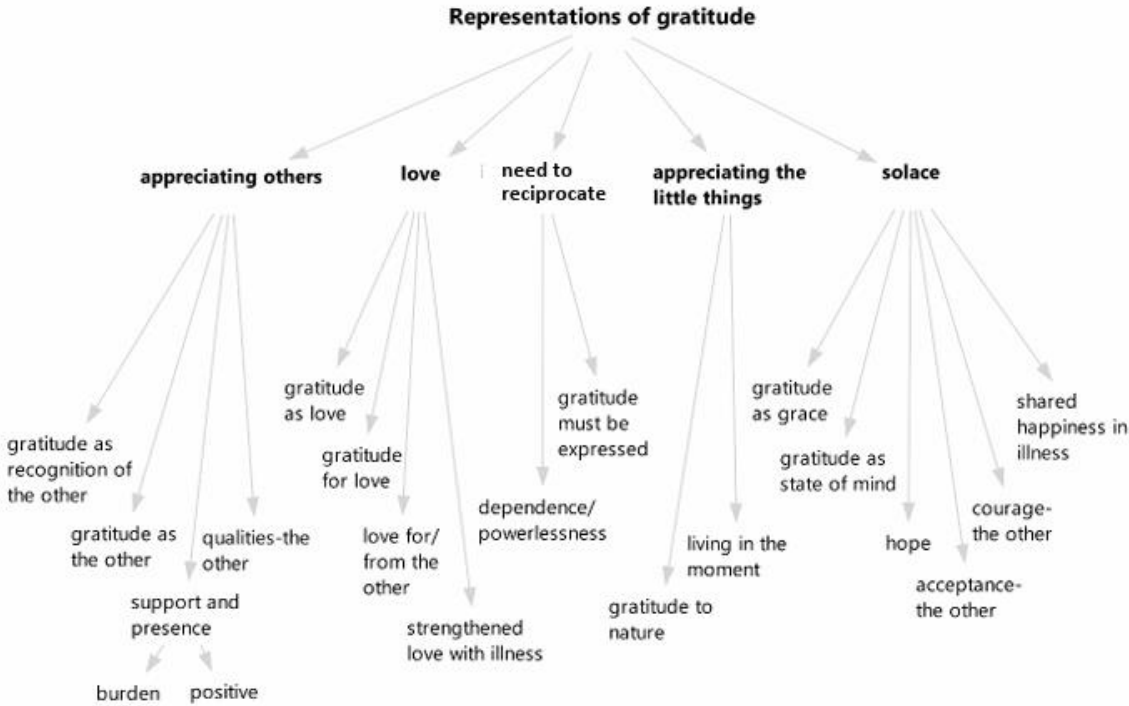

Figure S1. Coding tree of participants' representations of gratitude

Supplement: sj-pdf-1-pmj-10.1177_02692163231207495 – Supplemental material for ‘Thank you for loving me’: A qualitative study on perceptions of gratitude and their effects in palliative care patients and relatives [file sj-pdf-1-pmj-10.1177_02692163231207495.pdf]
