# Supplementary material for: ‘Thank you for loving me’: A qualitative study on perceptions of gratitude and their effects in palliative care patients and relatives
Source: Palliat Med. 2023 Nov 9;38(1):110–20. doi: 10.1177/02692163231207495 (PMC10798025; doi:10.1177/02692163231207495)
Supplement: sj-pdf-2-pmj-10.1177_02692163231207495 – Supplemental material for ‘Thank you for loving me’: A qualitative study on perceptions of gratitude and their effects in palliative care patients and relatives [file sj-pdf-2-pmj-10.1177_02692163231207495.pdf]

Supplementary material 2

Comparison of original French quotes with their English translations

| Original quote | English translation |
|---|---|
| **1. Appreciating other people** | |
| là tout le temps pour moi | "here for me at all times" (Z081) |
| d'être aussi chouette | "being so great" (X015) |
| la reconnaissance de ce que peut apporter l'autre | "recognition of what the other can bring" (X015-relative) |
| la gratitude finale, pour moi, c'était [mon épouse]. | "the ultimate gratitude, for me, it was [my wife]" (Z159) |
| ils nous ont soutenu […] au quotidien | "supporting us […] everyday" (X015-relative) |
| Je ne sais pas comment te remercier pour tout le bien que tu m'apportes, et ta gentillesse et ta bonté. | "I don't know how to thank you for all the good that you have done me, and your kindness and your goodness" (Y039-letter) |
| tous les taches [*sic*] autour la maison | "all the tasks around the house" (Y042-letter) |
| elle [mon épouse] est le rayon de soleil dans le noir de la maladie, sans sa présence ma vie est morne | "she [my wife] is the ray of sun in the darkness of the illness, without her presence my life is dull," Y037-letter |
| j'ai accepté ma maladie | "acceptance" of the illness (Y008) |
| après l'opération, c'était mes parents qui ont tout d'un coup été à côté de mon lit pour me prendre la main […] donc c'était, ouais, c'était un bon sentiment pour moi, même avec toutes les douleurs | "after the operation, it was my parents that were there all of a sudden at my bedside to hold my hand […] so it was, yeah, it was a good feeling for me, even with all the pain," Z067 |
| Si demain je meurs à cause de cette maladie, quelque part je serais en paix avec moi-même parce que j'ai remercié ma mère […] cette verbalisation a été vraiment salutaire | "If I die tomorrow because of this illness, somehow I will be at peace with myself because I thanked my mother […] verbalising was really salutary" (X015) |
| Je peux pas donner grand-chose en retour | "can't give much back in return" (Y037) |
| Je connais ta fatigue, je sais le temps que tu passes à prendre soin de moi. Je sais que je suis source d'inquiétude pour toi et, même épuisée, que ce soit physiquement ou moralement, tu es là. Pour moi. Uniquement pour moi. | "I know how tired you are, I know how much time you spend taking care of me. I know I am a source of worry for you and, even when you are exhausted, whether physically or morally, you are here. For me and only me." (Z057-letter). |

| | |
|---|---|
| tout ce qu'on peut s'amener comme soutien, comme toutes ces belles choses qui se passent d'humain à humain | "all the support we can bring each other, all those beautiful things that happen between humans" (Z172-relative). |
| **2. Love** | |
| Merci de m'aimer | "Thank you for loving me" (Y029-letter) |
| un sentiment qui est extraordinaire. Très proche de l'amour | "an extraordinary feeling. Very close to love" (Z144-relative) |
| un acte d'amour | "an act of love" (X025) |
| c'est surtout déjà d'aimer la personne | "above all to love the person" (Y037). |
| la maladie nous a rapproché encore plus l'un de l'autre […] et nous a démontré qu'on s'aime vraiment | "brought us even closer to each other […] and showed us that we really love each other" (Z081-letter) |
| La maladie s'est abattue sur toi, sur nous, sans crier gare, chamboulant tout, rendant tout futile, accessoire, dénudé d'intérêt, tout sauf une chose essentielle : l'Amour. L'amour que ces terribles circonstances ont, à mes yeux, renforcé au fil des terribles épreuves morales et physiques que tu as surmontées et auxquelles tu fais face aujourd'hui encore, tellement courageusement. | "The illness has befallen you, us, without any warning, turning everything upside down, rendering everything futile, superfluous, without any interest, everything but one essential thing: Love. The love that these terrible circumstances have, to my eyes, strengthened throughout these terrible moral and physical challenges that you overcame and that you are still facing today, so bravely" (X015-relative-letter). |
| un trésor | a "treasure" (Z057-letter) |
| Qu'aurais-je fait au milieu de tous ces dangers sans ton amour et ta sagesse ? | "What would I have done in the midst of all these dangers without your love and your wisdom?" X009-letter |
| je suis en train de m'accrocher et je veux continuer encore le plus longtemps possible d'être avec vous et à être aimée de vous | "I am hanging on and I want to continue for the longest time possible to be with you and to be loved by you," Y043-letter |
| **3. Need to reciprocate** | |
| De dire « merci » | "saying "thank you"" (Z272; Y042) |
| remerciement par le geste autant que par la parole. | "thanking through gestures as much as through words" (X015). |
| INT: est-ce que [la gratitude] c'est quelque chose dont vous aviez forcément déjà conscience ?<br>PRO: Non, pas vraiment.<br>PAT: Moi si. […]<br>PRO: Évidemment mon mari est dépendant de moi, donc…<br>PAT: T'aurais pu me mettre… n'importe où | Interviewer: Was it [gratitude] something you were already aware of [before the intervention]?<br>Relative: No, not really<br>Patient: I was […]<br>Relative: Of course my husband depends on me, so…<br>Patient: You could have placed me… anywhere (X009) |

| | |
|---|---|
| j'exprime ma gratitude […] je le fais maintenant parce que je suis handicapé si vous voulez. Alors tout qui m'arrive c'est la gratitude | "I express my gratitude […] I do it now because I'm handicapped, if you will. So everything that happens to me is gratitude" (Z159). |
| Depuis lors, j'ai vraiment appris la signification du mot "merci", et de la gratitude. Ce n'est pas toujours facile car j'ai eu l'habitude de me débrouiller seul, de décider seul dans ma vie professionnelle et puis, d'un seul coup, du jour au lendemain, je deviens dépendant de toi, des enfants, des médecins, des infirmières, des médicaments, des soins... | "since then [the illness], I really learned the signification of the words "thank you" and of gratitude. It's not always easy as I was used to manage alone, to decide alone in my professional life and suddenly, overnight, I become dependent on you, on the kids, the doctors, nurses, medicine, care…" (X015-letter) |
| INT: Et puis est-ce que vous avez l'habitude de l'exprimer votre sentiment de reconnaissance ou une gratitude […]?<br>PRO: *(rigole)* pas besoin *(rigole)*… non ben, ça fait partie…<br>PAT: Tu parles de qui toi ? Tu me dis « j'ai fait ça, je suis pas obligé de le faire… »<br>PRO: Non, c'est vrai. Il y a des fois je lui fais quand même remarquer certaines choses. […] Je lui dis « mais rends-toi compte que ça je ne suis pas obligée de faire » mais je le fais parce que j'aime mon mari et puis que je veux qu'il soit le mieux possible. | Interviewer: And do you usually express your feeling of gratefulness or gratitude […]?<br>Relative: (laughs) No need (laughs)… No, well, it's part of…<br>Patient: Who are you taking about? You tell me "I did this, I don't have to do it…"<br>Relative: No, it's true. Sometimes I point certain things out to him [my husband] […] I tell him: "You have to realise that I am not obliged to do that" but I do it because I love my husband and I want him to feel as good as possible. (X009) |
| de le remercier aussi du mieux qu'on peut, disons, en chargeant pas trop l'autre […] Je pense c'est aussi une forme de gratitude d'essayer de faire que l'autre puisse aussi se sentir bien. | "thanking also as best we can, let's say, not burdening the other too much […] I think that it's also a form of gratitude, to try to make the other feel good as well" (Z057-relative). |
| **4.Appreciating the little things** | |
| les longues "babillées" au téléphone | "the long chats on the phone" (X025-letter) |
| quand […] je vois ce paysage mais… voilà je suis pleine de gratitude | "when I […] see this landscape, well… I am filled with gratitude" (Z057-relative) |
| un petit bien-être par ci par là | "some wellbeing here and there" (Z057) |
| J'ai beaucoup de chance (Y037)<br>Je me rends compte de la chance que j'ai (X025-letter) | made them feel "lucky" (Y037; X025-letter) |
| y a… ah des bouffées comme ça de (souffle)… de bonheur qui viennent de je sais pas où. Et je pense que c'est en lien avec la gratitude. Quand je rentre dans | "There are… ah breaths of (exhales)… of happiness that come from I don't know where. And I think that it's linked with gratitude. When I enter my garden or I go |

| | |
|---|---|
| mon jardin ou que je vais me promener dans la nature, je sens un tel bien-être que j'ai l'impression qu'il y a un échange. | stroll in the countryside, I feel such a sense of wellbeing that I have the impression that there's an exchange." (X015-relative) |
| Chaque jour est un joli miracle que je savoure auprès de toi: MERCI !!! | "Every day is a pretty miracle that I savour next to you: THANK YOU" (Z080-relative-letter) |
| en mettant la barre un petit peu plus bas | "setting the bar a little lower" (Z172) |
| rien que de pouvoir descendre de son lit, de pouvoir s'habiller tout seul, se doucher, se préparer, rencontrer du monde […] tout ça, pour moi, c'est des raisons d'être reconnaissante | "just being able to get out of bed, get dressed without help, shower, get ready, meet people […] all these, to me, are reasons to be grateful" (Z172) |
| d'avoir de la gratitude ça remonte aussi le moral et puis je pense que si on prenait tous un petit peu plus la peine d'y réfléchir, ben peut-être qu'on arriverait à améliorer notre qualité de vie.[…] il faut se raccrocher à chaque petite chose qui peut nous montrer que ça vaut encore la peine et c'est ce que je me suis dit aussi quand j'ai appris, à priori, ce que j'ai c'est incurable | "to be grateful also helps cheer you up and I think that if we all made a little more effort to think about it, well maybe we would manage to improve our quality of life. […] we must hang on to every little thing that can show us that it's still worth it and that's what I told myself also when I learned that, on the face of it, what I have is incurable" (Z172) |
| **5. Solace in the midst of serious illness** | |
| une sorte de grâce | "a sort of grace" (Z159-relative) |
| vivre ensemble le mieux possible et… et de prendre les choses, on essaie, positivement | "live together as best we can and… and to take things, we try, positively" (X009-relative) |
| La manière dont tu surmontes la maladie me donne aussi une raison de te remercier | "the way in which you overcome the illness also gives me a reason to thank you" (X015-relative-letter) |
| Merci de me faire partager dans le quotidien difficile que la maladie t'impose, de doux moments de tendresse et d'Amour, de jolis sourires et des éclats de rires, des délires qui n'appartiennent qu'à nous. | "Thank you for sharing in the everyday difficulties that the illness is imposing on you, sweet moments of tenderness and Love, pretty smiles and bursts of laughter, a craziness that belongs to us alone." (Z080-relative-letter) |
| Je te remercie aujourd'hui d'avoir accepté de te battre, de lutter contre ce cancer afin que nous ayons encore de belles années devant nous | "Today I thank you for accepting to fight, to combat this cancer so that we can still have beautiful years before us," (Z057-relative-letter) |
| Tu acceptes mieux que moi ce qui t'es arrivé, de telle sorte que t'aider n'est pas pour moi un devoir, mais donne un sens à ma vie. | "you are accepting what happened to you better than I do, in such a way that helping you isn't a duty for me, but gives meaning to my life." (Z008-relative-letter) |
